# Non-*Aspergillus* Hyaline Molds: A Host-Based Perspective of Emerging Pathogenic Fungi Causing Sinopulmonary Diseases

**DOI:** 10.3390/jof9020212

**Published:** 2023-02-06

**Authors:** Samantha E. Jacobs, Thomas J. Walsh

**Affiliations:** 1 Division of Infectious Diseases, Department of Medicine, Icahn School of Medicine at Mount Sinai, New York, NY 10029, USA; 2 Center for Innovative Therapeutics and Diagnostics, 6641 West Broad St., Room 100, Richmond, VA 23220, USA

**Keywords:** *Fusarium*, *Scedosporium*, *Lomentospora prolificans*, *Scopulariopsis*, *Trichoderma*, *Paecilomyces*, *Acremonium*, *Rasamsonia*, *Arthrographis*, hyaline molds

## Abstract

The incidence of invasive sino-pulmonary diseases due to non-*Aspergillus* hyaline molds is increasing due to an enlarging and evolving population of immunosuppressed hosts as well as improvements in the capabilities of molecular-based diagnostics. Herein, we review the following opportunistic pathogens known to cause sinopulmonary disease, the most common manifestation of hyalohyphomycosis: *Fusarium* spp., *Scedosporium* spp., *Lomentospora prolificans*, *Scopulariopsis* spp., *Trichoderma* spp., *Acremonium* spp., *Paecilomyces variotii*, *Purpureocillium lilacinum*, *Rasamsonia argillacea* species complex, *Arthrographis kalrae*, and *Penicillium* species. To facilitate an understanding of the epidemiology and clinical features of sino-pulmonary hyalohyphomycoses in the context of host immune impairment, we utilized a host-based approach encompassing the following underlying conditions: neutropenia, hematologic malignancy, hematopoietic and solid organ transplantation, chronic granulomatous disease, acquired immunodeficiency syndrome, cystic fibrosis, and healthy individuals who sustain burns, trauma, or iatrogenic exposures. We further summarize the pre-clinical and clinical data informing antifungal management for each pathogen and consider the role of adjunctive surgery and/or immunomodulatory treatments to optimize patient outcome.

## 1. Introduction

During the past several decades, substantial progress in medical therapeutics in the fields of oncology, transplantation, and autoimmune/chronic inflammatory disease has led to an increased number of immunosuppressed patients with longer life expectancy. Many such patients are at risk for invasive mold diseases. The major portal of entry is the sinopulmonary tract, causing sinusitis and pneumonia. Less often, direct inoculation may occur via impairments in cutaneous or mucosal integrity due to, for example, central venous catheters or gastrointestinal ulceration. While *Aspergillus* spp. are the primary mold pathogens, non-*Aspergillus* hyaline molds are increasingly reported as causing superficial and invasive diseases due to improvements in molecular-based fungal diagnostics as well as the widespread use of antifungal prophylaxis that selects for these highly resistant fungi. This manuscript will review the relevant epidemiology, clinical manifestations, diagnosis, and management of the emerging hyalohyphomycoses causing sinopulmonary disease: *Scedosporium* spp., *Lomentospora prolificans*, *Fusarium* spp., *Scopulariopsis* spp., *Arthrographis kalrae*, *Trichoderma* spp., *Acremonium* spp., *Paecilomyces variotii, Purpureocillium lilacinum*, *Penicillium* spp., and *Rasamsonia* species. Often times, reviews of invasive fungal diseases are catalogued by organism. Another approach used herein to understand the epidemiology of these formidable pathogens in the context of immune impairment is host-based.

## 2. Taxonomy and Nomenclature of Clinically Relevant Species

The term “hyaline mold” refers to all septate molds that have hyaline or nonpigmented septate hyphae with acute angle branching in tissue and are distinguished in culture by their macroscopic and microscopic morphology. Improvements in molecular-based fungal diagnostics and phylogenetic analysis have triggered changes in fungal taxonomy so that certain fungi are newly recognized as distinct species. Described herein are the clinically relevant hyaline mold species that have been reported to cause invasive sinopulmonary mycoses in humans. A more detailed description of the taxonomy and nomenclature of the hyaline molds may be found in the *Atlas of Clinical Fungi* [1].

Worldwide, there are at least 200 *Fusarium* species grouped into approximately 10 phylogenetic species complexes [2]. The majority of human infections are due to members of the *F. solani* species complex and *F. oxysporum* species complex [3,4]. However, in an epidemiological survey of clinical specimens in Europe from 2007 to 2012, *Fusarium verticillioides* and *F. proliferatum*, belonging to the *Fusarium fujikuroi* species complex, were the most common isolates identified [5].

The nomenclature of the genus *Scedosporium/Pseudallescheria* has undergone several changes over the past decade. The genus *Scedosporium* now contains 10 species of which the *Scedosporium aurantiacum* and *Scedosporium apiospermum* species complexes (comprising five distinct species) are the most clinically relevant [6]. Previously known as *Scedosporium prolificans*, *L. prolificans* was renamed as it was shown to be unrelated to *Scedosporium* [6].

The vast majority of *Paecilomyces* infections in humans are due *P. variotii* species complexes, comprising *P. brunneolus*, *P. dactylethromorphus*, *P. divaricatus*, *P. formosus*, and *P. variotii sensu stricto* [7]. In the last decade, phylogenetic analysis of the 18S ribosomal RNA gene revealed that *P. variotii* and *P. lilacinus* were not related. *P. lilacinus* was transferred to a new family Ophiocordycipitaceae, and a new genus was proposed, *Purpureocillium*. Hence, in the present manuscript, *Paecilomyces variotii* and *Purpureocillium lilacinum* will be considered separately [8].

The majority of human infections due to *Scopulariopsis* species are due to *S. brevicaulis*, followed by *S. gracilis* and *S. brumptii* [9,10]. Several species of *Trichoderma* have been identified as human pathogens with *T. longibrachiatum* accounting for up to one-half of cases [11]. Less often, *T. harzianum*, *T. koningii*, *T. viride*, *T. bissettii*, and *T. citrinoviridae* have been reported [11,12,13]. 

Three *Acremonium* species have been definitively implicated in human disease using molecular methods, *A. kiliense* (now *Sarocladium kiliense*), *A. egyptiacum*, and *A. strictum* [14,15]. Of the more than 300 species of *Penicillium*, only a few including *P. chrysogenum* and *P. citrinum* are known to cause infection in humans [16]. The genus *Arthrographis* contains at least 13 species, of which only *A. kalrae* has been isolated in clinical specimens (https://www.mycobank.org/page/Name%20details%20page/31980, accessed on 1 October 2022) [17].

The *Rasamsonia* species complex, previously classified as *Geosmithia* spp., comprises 12 species [18]. *Rasamsonia argillacea* is a very rare human pathogen that may commonly be misidentified as *Penicillium* or *Paecilomyces* spp. based on microscopic morphology alone [19].

## 3. Environmental Epidemiology: Geographic and Environmental Reservoirs

*Fusarium* species are widely distributed in soil and plants. Initially described as a plant pathogen and a source of toxin production, *Fusarium* species are increasingly recognized as a human pathogen causing superficial, locally invasive, or disseminated disease in humans depending on host immunity [20,21]. While *Fusarium* infection may occur year-round, cases tended to cluster during the rainy summer season, correlating with high spore counts in the outdoor air [22]. A study of the genetic diversity by O’Donnell et al. of the *Fusarium oxysporum* complex, which investigated the multilocus DNA sequence data and amplified restriction fragment length polymorphisms of 18 environmental isolates and 88 clinical isolates of infections from four continents, found that there was a recent dispersal of a geographically widespread clonal lineage of 70% of the clinical isolates [23].

*Fusarium* spp. may also be a nosocomial pathogen. *Fusarium* has been identified in hospital water distribution systems, leading to outbreaks of invasive infection in immunocompromised patients [24,25,26]. In one Texas hospital served by municipal water supply, *Fusarium* spp. were identified in 57% of samples from the water system. Diverse *Fusarium* spp. were isolated in patient rooms including 82% of shower drains, 99% of sink drains, 8% of shower heads, and 18% of sink faucet aerators as well as nursing stations. Furthermore, aerosolization of *Fusarium* spp. was demonstrated in 27% of air samples collected after running water for 35 min, suggesting a route of transmission for sinopulmonary disease. Eight of 20 patients with *F. solani* infection had genetically similar isolates matched to environmental (*n* = 2) or other patient isolates (*n* = 6) [26]. A genetic diversity analysis of *F. oxysporum* established a matching clonal lineage between isolates from infected patients and environmental isolates from three U.S. hospitals, further supporting a nosocomial origin of fusarial disease [23].

Contact lens wear has been increasingly recognized as a risk factor for mycotic keratitis. Outbreaks of *Fusarium* keratitis were described in 2005–2006 in association with the use of a specific multipurpose contact lens solution, ReNu with MoistureLoc (RML) [27,28]. Ultimately, it was determined that the source of the fusaria was the immediate environment of the individual users. Suboptimal lens hygiene and properties of RML that facilitated the growth of *Fusarium* during lens storage were invoked as contributing factors [29].

*Scedosporium* species are distributed worldwide and are mostly commonly found in temperate climates. As soil saprophytes, niches in the outdoor environment include brackish water and saltwater, sewage, nutrient-rich thick muds as well as poultry manure, and cattle and bat feces [30]. Due to its propensity for stagnant and polluted water, *Scedosporium* is reported as causing pulmonary, central nervous system (CNS), and/or disseminated infection in near-drowning victims with a mortality approaching 70% [31]. In addition, *Scedosporium* species are the most common cause of eumycotic mycetoma in North America, resulting, for example, from soil contamination during penetrating trauma or abrasions [32].

*Lomentospora prolificans* is most commonly isolated from soil and potted plants and is geographically restricted to hot and dry climates including Australia, the Iberian peninsula, California, and the Southern United States [32].

*Purpureocillium lilacinum* is a saprobic organism that is ubiquitous in soil and air [8]. Although a rare pathogen in humans, it has been isolated in hospital water distributions including sinks and showers and survives well on polyurethane catheters [33,34]. Outbreaks of fungal endophthalmitis and invasive disease due to contaminated sodium bicarbonate solution and skin lotion, respectively, have been described in immunocompetent and immunocompromised hosts [35,36].

*Trichoderma* spp., *Acremonium* spp., and *Arthrographis kalrae* are found worldwide, most commonly recovered from soil but also from air [14,17]. Due to their ability to antagonize pathogenic fungi in soil and plants and stimulate plant growth, some *Trichoderma* strains are used as biopesticides [37]. *Scopulariopsis* spp., *Paecilomyces* spp., and *Penicillium* spp. are widely distributed in nature and are commonly isolated from soil, plant debris, decaying organic matter, and food products. *Penicillium* spp. are commonly considered airborne contaminants of clinical specimens, and recovery from clinical samples should prompt investigation for other disease etiologies [12,38]. The *Rasamsonia argillacea* species complex is thermotolerant fungi that have been isolated from dry soil and hot environments [39].

## 4. Affected Hosts and Spectrum of Disease

The differential diagnosis of non-*Aspergillus* hyaline mold pathogens according to host factors and clinical manifestations is provided in Table 1. An understanding of the true incidence and prevalence of the pathogenic non-*Aspergillus* hyaline molds remains elusive due to challenges in diagnosis, low autopsy rates, and the lack of systematic reporting [40]. Furthermore, much of the data for ultra-rare pathogens (e.g., *Trichoderma* spp. and *P. variotii*) are derived from registries (e.g., FungiScope), which are subject to reporting and publication biases and lack a denominator. Nonetheless, the limitations of informational registries are offset by their ability to promote international collaboration and synthesize meaningful epidemiologic and management data for orphan diseases [41].

### 4.1. Neutropenic Patients

#### 4.1.1. Host Defense/Pathogenesis

Much of the understanding of host immune responses is derived from studies of *Aspergillus* species; however, some mechanisms have also been elucidated in pre-clinical and animal models of fusariosis and scedosporiosis. In immunocompromised hosts, the principle portal of entry of non-*Aspergillus* hyaline molds is the sinopulmonary tract. Inhaled conidia reach the pulmonary alveoli, respiratory bronchioles, and subsegmental bronchi and germinate to become hyphae, the tissue-invasive form of filamentous fungi, in those with impaired host defenses. Less often, conidia may initiate infection via direct inoculation into cutaneous wounds or central venous catheters and disseminate to multiple organs [42]. 

The innate host immune response is critical to the pathogenesis of invasive mold disease, making neutropenic patients particularly vulnerable. Neutrophils are the primary effector cells recruited by alveolar macrophages [43,44]. The absence of neutrophils allows unchecked proliferation of hyphae through the tissue planes including the walls of blood vessels. This leads to thrombosis and hemorrhage from rapid and extensive hyphal growth with scant inflammatory response [45]. Without neutrophil recovery, angioinvasion and dissemination to other organs via the bloodstream ensues. Corticosteroids in immunocompromised patients further impair pulmonary host defenses by inhibiting the phagocytosis of conidia by macrophages and disrupting cytokine immune regulation.

#### 4.1.2. *Fusarium* Species

Amongst the non-*Aspergillus* hyaline molds, *Fusarium* spp. are the most common pathogens in neutropenic patients, particularly those with acute leukemia and aplastic anemia [46,47,48,49,50]. In one series of 20 patients with hematologic malignancy and fusariosis, of whom 19 (95%) were neutropenic, the most common signs or symptoms were fever (75%) and shortness of breath (70%), followed by cough (25%), chest pain (25%), and hemoptysis (5%) [46]. Cutaneous lesions, pneumonia, and sinusitis are observed in 68 to 91%, 50 to 84%, and 27 to 71% of patients, respectively, and approximately 40–60% of patients will have positive blood cultures [47,48,49,51,52]. Although eye involvement is uncommon, fungal endophthalmitis due to hyaline molds including *Fusarium* spp. and *S. apiospermum* is observed in approximately one-quarter of patients with hematologic malignancy and visual symptoms, highlighting the importance of ophthalmology consultation in such cases [53]. These patterns of organ involvement are similar in pediatric patients, as described in a recent systematic literature review of 106 cases of invasive fusariosis (IF) in children published between 1973 and 2021 [54]. Of particular concern in neutropenic patients is the development of *Fusarium* toenail paronychia, which may invade local soft tissues and then disseminate hematogenously to other organs [51,55]. In patients with disseminated fusariosis, multiple cutaneous lesions typically appear as painful, erythematous papular or nodular lesions that may develop central necrosis. 

Although similar to invasive aspergillosis in many aspects, IF is distinguished by a lesser propensity for pneumonia and sinusitis and a greater propensity for metastatic skin lesions and positive blood cultures due to adventitious sporulation [52]. Compared to non-neutropenic patients with IF, neutropenic patients are more likely to have fever at diagnosis, skin lesions, fungemia, and multiorgan involvement [47,56]. The radiographic pattern of pulmonary infiltrates in neutropenic patients with IF is non-specific and similar to that of other angioinvasive hyaline molds. Most often, alveolar infiltrates or macronodules are observed on computerized tomographic (CT) scan imaging; an exception is that the halo sign is more common in invasive pulmonary aspergillosis [46,52,57].

Outcomes are overall poor in neutropenic patients with IF, particularly in the absence of neutrophil recovery. In a multicenter Spanish study of 58 patients with IF from 2000 to 2015, 90-day mortality was 29% versus 66% in the non-neutropenic patients versus patients who presented with neutropenia, respectively (*p* = 0.01) [56]. However, the mortality increased to 91.3% in patients with persistent neutropenia and improved to 38% amongst those with neutrophil recovery. Strikingly, in the largest series of pediatric patients with IF including 22 children from hematology-oncology departments in Israel, 17 (86%) patients were neutropenic, but the 90-day mortality was 23%, considerably lower than that observed in adults. However, all four patients who died with active fusariosis were neutropenic with relapsed/refractory acute leukemia, whereas neutrophil count recovery was noted in all of the surviving patients (*n* = 17) [58].

#### 4.1.3. *Scedosporium* Species

After *Aspergillus* and *Fusarium*, *Scedosporium* spp. are the most common hyaline molds causing sino-pulmonary disease neutropenic patients. As noted above, the *S. apiospermum* complex is the most common cause of scedosporiosis. In neutropenic patients and patients with hematologic malignancy, disseminated infection occurs in as much as 50%, with pulmonary disease being the most common (65%), followed by osteoarticular/skin (53%), fungemia (30%), and brain (24%) [59]. Signs and symptoms of pulmonary disease are non-specific and include fever, cough, dyspnea, and pleuritic chest pain, with the caveat that most case series do not distinguish amongst different host groups [32,60]. Mortality is higher in patients with hematologic malignancies (up to 70%) compared to other immunocompromised hosts; however, lower mortality rates have been observed in more recent years with the increased use of voriconazole rather than amphotericin B, to which *S. apiospermum* has intrinsic resistance [61].

#### 4.1.4. *Lomentospora prolificans*

Invasive disease due to *L. prolificans,* often classified as a breakthrough infection, is particularly ominous in neutropenic patients due to the organism’s intrinsic high-level of antifungal resistance. The clinical presentation of invasive lomentosporiosis differs from that of scedosporiosis in that neutropenia, dissemination, and fungemia are significantly more common in the former [59,62]. Indeed, nosocomial outbreaks of disseminated lomentosporiosis due to hospital renovation and/or absent HEPA filters have been described in 10 patients with acute leukemia with 100% fatality. All patients had positive blood cultures and nine (90%) had pneumonia [63,64]. On chest radiography, six patients had bilateral pulmonary infiltrates and three had pleural effusions [63,64]. 

Other sites of organ involvement in neutropenic patients with *L. prolificans* infection include osteoarticular, skin, brain, and heart [59]. From the FungiScope registry, of 41 patients diagnosed with invasive lomentosporiosis between 2008 and 2019, 27 (66%) patients had underlying malignancy, although the number of neutropenic patients was not specified [65]. Attributable mortality was 77% within this cohort. 

#### 4.1.5. *Scopulariopsis* Species

*Scopulariopsis* is a rare but devastating pathogen due to its intrinsic resistance to nearly all available antifungal agents. In neutropenic patients, manifestations of *Scopulariopsis* include sinusitis, mastoiditis, pneumonia, brain abscess, and locally invasive soft tissue infection [66,67,68,69,70]. Due to its rarity, the radiographic appearance of pulmonary disease is not well-characterized, but dense lobar consolidation and cavitary lesions have been described in case reports [70,71]. Surgical resection of localized infection when feasible and restoration of innate host immunity are critical. Indeed, invasive infection in immunocompromised hosts is almost always fatal.

#### 4.1.6. *Acremonium* Species

Invasive disease due to *Acremonium* species is rare but increasingly reported due to improvements in fungal diagnostics. The vast majority of infections occur in immunocompromised hosts, particularly patients with hematologic malignancy and hematopoietic cell transplant (HCT) recipients. The organism may be introduced via the respiratory tract or central venous catheters [72,73]. Amongst the neutropenic patients, the clinical features mirror that of *Fusarium* in that fever and disseminated skin papules predominate, and blood cultures are frequently positive [73,74,75,76,77,78,79,80] Overall, pulmonary involvement is less common compared to other hyaline molds [81]. When present, multiple nodules with or without cavitation are observed on chest imaging in neutropenic patients [78,79]. Additionally, similar to *Fusarium*, mortality is high in the absence of neutrophil recovery. 

#### 4.1.7. *Trichoderma* Species

Fifty invasive infections due to *Trichoderma* species were identified in the FungiScope registry and published literature through December 2021. Hematologic malignancy was the most common underlying condition (*n* = 19, 38%), and 14 (28%) patients were neutropenic [11]. In six individual case reports of invasive *Trichoderma* infections in neutropenic hosts, the lungs were most commonly involved. Chest CT demonstrated solitary mass-like lesions with cavitation and multiple pulmonary nodules with or without a halo sign [82,83,84,85,86,87]. 

#### 4.1.8. *Paecilomyces variotii* and *Purpureocillium lilacinum*

Analyses of cases of invasive fungal disease due to *P. variotii* (56 cases including 43 proven and 16 probable) and *P. lilacinum* (101 cases including 85 proven and 16 probable) from the FungiScope registry and literature review were recently published to provide significant insights into the epidemiology and management of these pathogens [88,89]. Amongst patients with *P. variotii* infection, the most common underlying conditions were hematologic malignancy and peritoneal dialysis. Nine (15%) patients with *P. variotii* were neutropenic at diagnosis, primarily due to hematologic malignancy. All nine patients presented with fever. Sites of organ involvement were lung (*n* = 6), blood (*n* = 1), central nervous system (*n* = 1), and disseminated (*n* = 1). Four (44%) patients died [88]. Amongst the *P. lilacinum* cases, there were 14 (14%) neutropenic patients, of whom five had solid tumors. Disseminated disease was common with positive blood cultures in six (43%) patients, a finding that may be related to its propensity to sporulate (adventitious sporulation) in tissue, similar to that observed in *Fusarium* spp. and *Acremonium* spp. [90]. Five neutropenic patients had isolated sinopulmonary disease, and one patient had cutaneous infection. 

#### 4.1.9. Other Non-Aspergillus Hyaline Molds

Although *Arthrographis* has been reported as a pulmonary pathogen in immunocompromised hosts, to our knowledge, there have been no published cases of invasive disease in neutropenic patients. *Rasamsonia* spp., one of the most rare human pathogens amongst the hyaline molds, are primarily described in patients with chronic granulomatous disease [91].

### 4.2. Hematopoietic Cell Transplant and Solid Organ Transplant Recipients

#### 4.2.1. Host Defense and Pathogenesis

Both HCT and solid organ transplant (SOT) recipients are at risk for invasive mold diseases due to severe T-cell immunodeficiency, associated with underlying disease and/or iatrogenic immunosuppression, particularly corticosteroids. In post-engraftment HCT recipients, immune dysregulation due to graft-versus-host disease (GVHD), and its treatment are additional risk factors. Corticosteroids impair the killing of *Aspergillus* conidia by interfering with phagocytosis, intracellular oxidative burst, production of cytokines and chemokines, and cellular migration [92]. Other treatment and prevention strategies of GVHD including the use of calcineurin inhibitors, alemtuzumab, and JAK-STAT inhibitors may also impair the host response to invasive fungal pathogens. Additional factors such as co-infection with cytomegalovirus may contribute to further immune dysregulation in HCT and SOT recipients [93]. Polymorphisms in genes encoding innate host defense molecules such as TLR4 and dectin may also increase the risk of the development of invasive mold diseases [94,95]. While these human genetic studies have addressed the increased risk of pulmonary aspergillosis, one can reasonably hypothesize, as a general principle, that polymorphisms in innate host defense molecules may also increase the propensity for the development of invasive fungal disease caused by the less common hyaline molds discussed herein. Although their impact on the pathogenesis of *Fusarium*, *Scedosporium*, and other rare molds has not been formally assessed, parallels may be drawn to *Aspergillus*, where patterns of tissue injury are host-dependent [45].

In non-neutropenic hosts, an exacerbated inflammatory response characterizes invasive disease due to *Aspergillus* spp., rather than uncontrolled hyphal growth and angioinvasion in neutropenic patients. This coincides with the clinical observation that invasive disease due to hyaline molds is more likely to be disseminated in neutropenic patients compared to transplant recipients. Among the lung transplant recipients, there are additional unique factors that make such hosts particularly vulnerable to invasive pulmonary disease: direct communication between the transplanted organ and the external environment, impaired mucociliary clearance, and a vulnerable anastomotic site.

#### 4.2.2. *Scedosporium* and *L. prolificans*

In the transplant population, scedosporiosis usually occurs as a breakthrough infection [96]. Patients in this study presented with pulmonary scedosporiosis. Pulmonary disease data from the Transplant-Associated Infection Surveillance Network found that *S. apiospermum* species complex and *L. prolificans* accounted for 13% of non-*Aspergillus* mold infections in the HCT population between 2001 and 2006, following Mucorales and *Fusarium* species [97]. In contrast, *Scedosporium* spp. and *L. prolificans* represented approximately 25% of non-*Aspergillus* mold infections amongst the SOT recipients.

Husain et al. described the clinical characteristics of infections due to *S. apiospermum* and *L. prolificans* in 80 SOT and HCT recipients identified at their institutions or in the published literature. Of the 23 HCT recipients, 67% were neutropenic and 52% had GVHD [98]. The clinical presentation was similar between the HCT and SOT patients, with pulmonary infection being the most common (42%), followed by skin (33%), and CNS (32%). Disseminated disease and fungemia were more common in the HCT (69% and 33%, respectively) than SOT recipients, (46% and 11%, respectively), perhaps reflecting a greater degree of immune impairment in the former. Less common clinical manifestations in transplant recipients include sinusitis, endophthalmitis, endocarditis, mycotic aneurysm, renal abscesses, septic arthritis and osteomyelitis, gingival abscess, and cystitis [53,61,96,98].

Chest imaging, as described in heart and lung transplant recipients with scedosporiosis, may show hilar lymphadenopathy and bilateral tree-in-bud nodules that progress to noncavitary nodular consolidations [99,100]. Surrounding ground glass (“halo sign”) is variably present.

Overall mortality is approximately 50% in the HCT and SOT recipients with scedosporiosis and lomentosporiosis, with the highest rates observed in HCT recipients with disease due to *L. prolificans* [61,98]. In univariate analyses, disseminated infection, CNS involvement, fungemia, and renal failure were all associated with higher mortality whereas adjunctive surgery and receipt of voriconazole (for disease due to *Scedosporium* spp.) were associated with lower mortality [61,98].

#### 4.2.3. *Fusarium* Species

The frequency of sino-pulmonary and disseminated *Fusarium* infection is 1–4% in allogeneic HCT recipients, for whom it is the third most common mold infection, following *Aspergillus* and Mucorales [47,97,101]. Consistent with the observation that invasive fusariosis most commonly affects neutropenic hosts, approximately half of the HCT recipients with *Fusarium* infection are reported to have neutropenia, and 22 to 57% have acute GVHD [97,102]. Nucci and colleagues analyzed 61 cases of fusariosis in allogeneic HCT recipients from the United States and Brazil [102]. The median time from HCT to diagnosis of fusariosis was 48 days, and the majority of patients had disseminated infection with metastatic skin lesions (75%). In a prospective cohort study including 663 HCT recipients from eight centers in Brazil in which 17 cases of IF were diagnosed, risk factors for IF were receipt of antithymocyte globulin, hyperglycemia, and history of AML in the early phase (prior to day 40) HCT recipients, and nonmyeloablative conditioning regimen, grade III/IV GVHD, and prior invasive mold disease in the late-phase HCT recipients [47].

*Fusarium* infection is extremely rare in SOT. A recent review of the literature identified 26 cases since 1998 including 10 lung transplant recipients [103]. Pneumonia is the most common manifestation of IF in lung transplantation, again with a clinical and radiographic presentation that mirrors that of invasive pulmonary aspergillosis. Myriad radiographic findings have been reported in lung transplant recipients with Fusarial pneumonia: abscesses, consolidation, nodular opacities, ground glass opacities, pleural effusions, and bronchiectasis [103,104]. In contrast, locally invasive cutaneous infection predominates in non-SOT recipients [103,105]. Other reported manifestations in SOT are osteomyelitis, endocarditis, and fungemia.

#### 4.2.4. *Paecilomyces variotii* and *Purpureocillium lilacinum*

As noted above, *P. variotii* and *P. lilacinum* are rare pathogens in humans, and thus there are relatively few reported cases in transplant recipients. However, from the analyses of the FungiScope registry, some patterns are evident. Whereas prosthetic valve endocarditis and peritoneal dialysis catheter-associated peritonitis are the most common in immunocompetent hosts, HCT and SOT recipients with *P. variotii* infection most often present with pneumonia [88]. In two allogeneic HCT recipients, chest CT demonstrated patchy consolidative opacities or ground glass nodules [106,107]. Sinopulmonary disease is also the most common in HCT and lung transplant recipients with *P. lilacinum* infection including the involvement of the bronchial stent in the latter group [89]. The vast majority of non-lung SOT recipients with *P. lilacinum* have localized skin or deep soft tissue infection. A variety of skin lesions are described in the SOT population including erythematous or violaceous nodules, ulcerated plaques, and cellulitis [108].

#### 4.2.5. Other Non-Aspergillus Hyaline Molds

Invasive fungal disease due to *Scopulariopsis* spp. and *Rasamsonia* spp. are primarily reported in HCT where disseminated infection predominates, followed by isolated pulmonary involvement [66,91,109,110]. In contrast, from the FungiScope registry and literature review, the clinical presentations of *Trichoderma* diseases are varied in transplant recipients: pneumonia, mediastinitis, endocarditis, fungemia, and peritonitis [11]. There is a paucity of reports of invasive fungal disease due to *Acremonium* spp., *Arthrographis kalrae*, and *Penicillium* spp. in transplant recipients.

### 4.3. Chronic Granulomatous Disease

#### 4.3.1. Host Defenses and Pathogenesis

Among the primary immunodeficiencies that increase the risk for the development of invasive pulmonary mold diseases, chronic granulomatous disease (CGD) is the most prevalent [111,112]. Chronic granulomatous disease is a genetic disorder of the nicotinamide adenine dinucleotide phosphate (NADPH) oxidase complex, so that phagocytes have impaired production of the microbicidal reactive oxidant superoxide anion and its metabolites [113]. The disease, which is frequently diagnosed during childhood, is characterized by recurrent life-threatening bacterial and fungal infections, although mortality has improved in recent years with the adoption of routine antimicrobial prophylaxis and the administration of interferon-gamma [113]. The pathogenesis of invasive mold disease in this condition is best described in a murine model of CGD and *A. fumigatus* infection [114]. Lung histology in the animal model demonstrates pyogranulomatous lesions and invasive hyphae, but no hyphal angioinvasion or coagulative necrosis [114], which parallels the histopathological findings in patients with CGD [45]. Although the pathogenesis of non-*Aspergillus* hyalohyphomycetes in CGD is not well-characterized, a similar exuberant inflammatory response is thought to be the direct cause of pathology, as opposed to uncontrolled fungal growth with angioinvasion and dissemination in neutropenic patients.

#### 4.3.2. Rasamsonia Species and Other Molds

In patients with CGD, the vast majority of hyaline mold infections manifest as pneumonia; extrapulmonary involvement is rare [115,116]. *Rasamsonia* spp., primarily *R. argillacea*, are emerging fungal pathogens that, in retrospect, appear to be the most common cause of non-*Aspergillus* invasive fungal diseases in patients with CGD [117,118,119]. *R. argillacea* may present as a breakthrough infection due to its intrinsic resistance to voriconazole, which is often administered for antifungal prophylaxis. Through microscopic morphology, *Rasamsonia* spp. may appear very similar to *Paecilomyces* spp., leading to the reassignment of misidentified pathogens once genomic sequencing was available and performed [118]. Thirteen cases of invasive *Rasamsonia* spp. infection are summarized in two literature reviews with a mortality rate of 42% [118,119]. The majority of patients had pulmonary involvement (*n* = 11), complicated by chest wall invasion (*n* = 4) and dissemination to the brain (*n* = 3). Radiographic pulmonary findings are described as nodules, abscesses, or infiltrates. *Scedosporium apiospermum* and *L. prolificans* are rarely reported as causing pulmonary abscesses with or without local chest wall invasion, brain abscess, and vertebral osteomyelitis in patients with CGD [120,121,122].

### 4.4. Acquired Immunodeficiency Syndrome

Overall, invasive mold disease is rare in persons with HIV/AIDS with the increased use of effective antiretroviral therapy (ART) in the past two decades. Prior to effective ART, *Aspergillus* was the most common invasive mold pathogen in this population, frequently observed as sino-pulmonary disease in patients with advanced HIV infection and associated neutropenia from disease or medication-induced myelosuppression (e.g., zidovudine) [123,124]. There is a paucity of reported cases of non-*Aspergillus* hyaline mold infections in persons with HIV. Such reports include invasive sinusitis (*S. apiospermum*), invasive sinusitis and meningitis (*A. kalrae*), disseminated fusariosis (*F. solani* and *F. verticillioides*), and necrotizing esophagitis (*P. chrysogenum*) [125,126,127,128]. In recent publications describing non-*Aspergillus* hyaline mold diseases identified via the FungiScope registry and PubMed literature review, HIV/AIDS was an underlying condition in a total of 12 patients (*P. lilacinum*, *n* = 4; *Trichoderma* spp., *n* = 2; *Scedosporium* spp., *n* = 6) [11,61,62,89]. Central venous catheter-related fungemia was the most common manifestation of *P. lilacinum* and *Trichoderma* spp. infection; the manifestations of scedosporiosis were not specified.

### 4.5. Cystic Fibrosis

Patients with cystic fibrosis (CF) are predisposed to fungal colonization of the airways due to impaired mucociliary clearance, development of bronchiectasis as well as exposure to prolonged antibiotic therapy and corticosteroids. *Scedosporium apiospermum* is the most common non-*Aspergillus* hyaline mold to affect patients with CF, followed by *S. aurantiacum* and *L. prolificans* [129]. In a prospective study of 128 patients with CF, the *S. apiospermum* species complex was isolated in bronchial secretions in 8.6% of patients, ranking third after *Candida* species (61%) and *Aspergillus fumigatus* (46%) [130]. In most cases, *S. apiospermum* colonization is asymptomatic, although allergic bronchopulmonary disease and rarely invasive disease have been described [130,131,132]. Patients with CF who receive corticosteroids are at increased of invasive pulmonary disease caused by colonizing mold pathogens. 

Of great concern in colonized patients with CF is the risk of invasive disease following lung-transplantation, an increasingly utilized therapy in those with end-stage lung disease. There are limited data evaluating the outcomes among patients with CF colonized with non-*Aspergillus* molds who subsequently undergo transplant. However, a recent single-center study in the era of mold-active triazole prophylaxis suggests that outcomes may be favorable for patients with *S. apiospermum* species complex colonization [133]. Among the eight CF patients colonized with the *S. apiospermum* species complex prior to transplant, none developed invasive *Scedosporium* infection post-transplant and seven survived more than one-year post-transplant.

*Scedosporium* spp. and *Lomentospora* spp. may also interfere with bacterial development in the lungs of patients with CF. Peptidorhamnomannans (PRMs) are glycoconjugates on the cell walls of *Scedosporium* spp. and *Lomentospora* spp., which facilitate adhesion to host epithelial cells and interact with the host immune system. In vitro data demonstrate that these PRMs from *L. prolificans*, *S. aurantiacum*, *S. boydii*, and *S. apiospermum* are capable of inhibiting bacterial growth, specifically that of methicillin-resistant *Staphylococcus aureus* and *Burkholderia cepacia* as well as biofilm formation [134]. Understanding the nature and mechanisms of fungal–bacterial interactions in the lungs of patients with CF may ultimately lead to novel therapeutics.

### 4.6. Immunocompetent Hosts

As opportunistic pathogens, non-*Aspergillus* hyaline molds may cause invasive infection in previously healthy individuals who sustain burns, trauma, near-drowning event, or in those with more subtle immune impairments caused by underlying conditions including malnutrition, alcohol abuse, or diabetes. Typically, the portal of entry is via catheters or direct cutaneous inoculation; therefore, sinopulmonary disease is less common in this host group. Locally invasive cutaneous fusariosis may develop in previously healthy patients in the setting of mechanical trauma and burn injury or may present as cellulitis, chronic ulceration, subcutaneous abscess, and plaques in the setting of preexisting skin breakdown [135]. 

As noted above, pulmonary, sinus, and CNS infection due to *Scedosporium* spp. are reported in victims of near-drowning in polluted waters [32]. The relation between near-drowning events and CNS scedosporiosis is characteristic for *Scedosporium* spp. among the other hyaline molds, *Aspergillus* spp., and the Mucorales [136]. The exact mechanism of disseminated infection in near-drowning victims is uncertain, but is thought to occur via hematogenous spread following massive inoculation of the organism into the respiratory tract during the aspiration of infested water or by translocation of organisms from contaminated water through the cribriform plate and adjacent sinuses. For those drowning victims who may have developed clinically occult disseminated scedosporiosis, there is the potential of donor organs being infected with *Scedosporium* spp. [137]. 

There are also case reports of fungal endocarditis in immunocompetent hosts due to non-*Aspergillus* hyaline molds related to injection drug use and/or prosthetic heart valves [138]. Finally, invasive disease may be iatrogenic in etiology. Contamination of peritoneal dialysis catheters leading to peritonitis or of central venous catheters causing fungemia is reported with *Trichoderma* spp., *Paecilomyces variotii*, and *Fusarium verticillioides* [139,140,141,142]. 

## 5. Diagnosis of Non-*Aspergillus* Hyaline Molds

### 5.1. Culture-Based Methods and Histopathology

The hyaline molds are distinguished in culture by their macroscopic and microscopic morphology. It was not within the scope of this review to provide a comprehensive comparative description of the morphologic features of the non-*Aspergillus* hyaline molds. However, it behooves the infectious diseases and pulmonary physician to achieve a basic understanding of the characteristics of these organisms in culture in order to inform management. Some distinctive features derived from [35] are described below. 

Sabouraud dextrose agar is the medium most commonly used in clinical microbiology laboratories to isolate fungi from clinical specimens. Special selective media such as SceSel+ have increased the recovery rate of *L. prolificans* in patients with CF [143]. The majority of the agents of hyalohyphomycoses grow rapidly within 5 days; exceptions are *Scedosporium* spp. and *Acremonium* spp., which mature in 5–7 days, and *Arthrographis kalrae* at 10–21 days. 

*Fusarium* species are identified by the presence of banana- or canoe-shaped, multicellular macroconidia in mature colonies. These colonies develop a pink or violet center with a lighter periphery, or, in the case of the *F. solani* species complex, a blue-green color where conidial cells form. *Fusarium* spp., in their early growth, display conidiophores and microconidia, which may be similar to those of *Acremonium* species, resulting in laboratory misidentification. As noted above, *Fusarium* and *Acremonium* as well as *P. lilacinum* demonstrate adventitious sporulation, and the identification of reproductive structures (conidiophores and conidia) in histopathology specimens or the direct examination of blood cultures may allow for provisional diagnosis of one of these pathogens [144]. Caution should be used in applying special stains such as the Fontana–Masson stain as the sole basis for distinguishing hyaline versus dematiaceous molds [145].

The microscopic morphology of *Penicillium* spp., *Rasamsonia* spp., and *P. variotii* is similar, but the phialides of *P. variotii* are more elongated and taper, giving them the appearance of drawn-out tenpins or tapering candles. Molecular and proteomic methods are best used for accurately distinguishing these species in clinical microbiology laboratories. The anamorph (asexual form) of *Talaromyces marneffei* may be morphologically similar to *Penicillium* species, but is distinguished by its thermal dimorphism and the presence of a diffusible red pigment.

*A. kalrae* is distinct amongst the hyaline molds due to its smooth yeast-like appearance during early growth. The colony surface is initially smooth and a cream to pale yellow color, and then later velvety, resembling a mold. Similarly, on the microscopic examination of tissue, yeast cells are seen in early growth, and septate hyaline hyphae are observed in later growth.

In the fluid and tissue histopathology specimens, the hyaline molds appear as nonpigmented acutely angled branching septate hyphae, which may resemble *Aspergillus* spp. [144]. Although culture or molecular-based techniques are required for definitive diagnosis, one may appreciate subtle differences in hyphal morphology between *Aspergillus* species and *Scedosporium* species. Whereas *Aspergillus* spp., *Scedosporium* spp., and *L. prolificans* display a pattern of dichotomous branching septate hyphae in tissue or on the direct exam of respiratory tract specimens, the advancing hyphal structure of *Scedosporium* may demonstrate terminal anneolloconidia [38].

Amphotericin B, triazoles, and echinocandins may alter the histological morphology of *Aspergillus* and non-*Aspergillus* hyaline molds to result in truncated, distorted, irregularly branched, and fragmented hyphal structures [146,147]. These histological appearances are different from the classical architecture of the well-formed acutely angled branching hyaline septate hyphae of hyaline molds. Thus, one should use caution in excluding a histological diagnosis of a hyaline mold infection based upon atypical histological structures in patients receiving antifungal therapy.

### 5.2. Molecular Diagnostics 

Culture-based methods are historically the gold standard for fungal diagnosis, though these methods are often time-consuming and lack sensitivity. Diagnosis of mold infection via molecular-based testing including nucleic acid amplification assays and mass spectroscopy is increasingly utilized, but not yet available at many medical centers [148]. Matrix-assisted laser desorption ionization/time-of-flight mass spectrometry (MALDI-TOF MS) is a powerful new tool that identifies cultured bacteria and fungi from clinical specimens [149]. MALDI-TOF is widely accepted for identifying yeasts. Although there are encouraging advances in the application of MALDI-TOF in the identification of common molds, there are still some limitations for filamentous fungi including a need to expand the reference library of commercial databases to facilitate accurate identification [150].

A broad range of panfungal PCR is increasingly employed in clinical laboratories to identify fungi directly from the fresh tissue or fluids (e.g., cerebrospinal fluid) as well as formalin-fixed paraffin-embedded (FFPE) tissue. Most often, the internal transcribed spacer (ITS) regions or the 28S rDNA locus are amplified using universal fungal primers, followed by DNA sequencing. Although this method is highly specific, the sensitivity is limited by specimen type, with the lowest yield from FFPE tissue [151,152]. Several PCR platforms including the multiplexed PCR/liquid-phase array, PCR/electrospray ionization mass spectrometry, and labeled primer PCR have demonstrated encouraging results in the detection of pulmonary fungal pathogens including hyaline molds [153,154,155]. Importantly, PCR should only be pursued for the detection of fungi from tissue samples when fungal elements or structures are identified in histopathology, and PCR is not recommended when fungal stains are negative [156].

A species-specific multiplex PCR assay has been developed to detect clinically relevant *Scedosporium* species and *L. prolificans* directly from the sputum specimens from CF patients [157]. rDNA ITS sequencing appropriately identifies the main species in *Scedosporium*, but the partial β-tubulin gene is required to differentiate closely related species [158]. For *Fusarium* spp., a PCR platform based on the intergenic spacer (IGS), rather than ITS regions, has been developed to facilitate speciation. At present, multi-locus sequence typing is currently favored for the identification of clinical *Fusarium* isolates to the species level [158,159].

As one looks toward the future developments of molecular diagnostics for invasive mold disease, a reasonable strategy would be to develop a multiplex qPCR system for the detection of the common fungal causes of pneumonia, which include the medically important molds: *Aspergillus* spp., Mucorales, *Fusarium* spp., *Scedosporium* spp., *Lomentospora prolificans*, *Purpureocillium lilacinum*, *Paecilomyces* spp., *Rasamsonia* spp., and *Scopulariopsis* species. Further consideration would be to include the pathogenic yeasts that also cause lower respiratory tract disease: *Cryptococcus neoformans* and the endemic dimorphic pathogens (*Histoplasma capsulatum*, *Blastomyces dermatitidis*, *Coccidioides* spp., *Talaromyces marneffei*, and *Emergomyces* spp.). Whether the primers for all of the medically important molds and yeasts causing lower respiratory tract disease could be feasibly combined without primer/dimer interference would determine in part the need to have one system or two systems for molds and yeasts. Bronchoalveolar fluid would be the most immediately practical matrix in which to develop such assays due in part to the relative ease of nucleic acid extraction and the amount of fungal DNA in a sample. In comparison, the development of the assay using blood as the matrix becomes more challenging due to the lower concentration of fungal DNA and challenges in the extraction procedure to remove inhibitors.

### 5.3. Fungal Biomarkers

The *Aspergillus* galactomannan (GM) enzyme-linked immunosorbent assay (EIA) is an important diagnostic tool for invasive aspergillosis in immunocompromised hosts [160]. Serum and bronchoalveolar lavage fluid (BALF) are the two most widely sampled specimens for GM EIA. The GM EIA may also demonstrate cross-reactivity with other hyaline molds including *Fusarium* spp., *Penicillium* spp., and *Paecilomyces* species [161]. This phenomenon was illustrated in a study of 18 patients with hematologic malignancy or HCT and invasive fusariosis, in which 15 (83%) patients had at least one positive serum GM [162]. Of note, (1→3)-β-D-glucan (BDG) is a component of the cell wall of *Scedosporium* species, *L. prolificans*, *Fusarium* spp., and many other fungi, and consequently, may be elevated in serum and BALF during pneumonia and in serum during disseminated infection. However, due to its limited specificity, BDG cannot reliably be used for definitive diagnosis.

## 6. Management of Sino-Pulmonary Hyalohyphomycoses

### 6.1. Establishing Infection Versus Colonization

When there is growth of a hyaline mold from a sterile site (e.g., blood or tissue, or histopathologic evidence of invasive disease), directed treatment should be initiated. Establishing the pathogenicity of non-*Aspergillus* hyaline molds when isolated in culture from non-sterile specimens can be more challenging (e.g., BALF, sputum, or sinus aspirate), particularly in patients who are mildly immunosuppressed or who have atypical signs/symptoms. For example, *Scedosporium* spp. commonly colonize the airways of patients with CF and chronic bronchiectasis. Patients with solid tumors are another host group with mild to moderate immunosuppression in whom the detection of saprophytic molds in respiratory samples rarely has clinical significance [163]. When non-*Aspergillus* hyaline molds are identified in these non-sterile specimens, one must consider the clinical evidence to support invasive disease (e.g., patient symptoms, radiographic pulmonary infiltrates, as well as the underlying host immune status) when making the decision to treat. An exception is *Penicillium* spp. which is such a rare cause of hyalohyphomycoses that its isolation in culture should always prompt investigation for other pathogens.

### 6.2. Role and Interpretation of Antifungal Susceptibility Testing

The antifungal susceptibility pattern of the non-*Aspergillus* hyaline molds may be highly variable for some fungi (e.g., *Fusarium* spp.) and more predictable for other pathogens such as the *Scedosporium* species. Notably, there are no established clinical breakpoints for the non-*Aspergillus* hyaline molds using the Clinical Laboratory and Standards Institute (CLSI) or European Committee on Antimicrobial Susceptibility Testing (EUCAST) methodology. Recent global guidelines recommend susceptibility testing for epidemiologic purposes [164]. Due to the lack of interpretive breakpoints, antifungal susceptibility determinations for treatment must be interpreted cautiously. Nucci and colleagues reported that MICs by the CLSI or ECAST methods did not correlate with therapeutic response in patients with invasive fusariosis, while the recovery from neutropenia was the critical determinant for success outcome [165]. Moreover, Navarro-Rodríguez and colleagues observed that there was no therapeutic response in experimental disseminated murine fusariosis produced by organisms with MICs within the epidemiological cut-off values [166]. Clearly, more work is needed in developing improved in vitro methods that may predict the outcome. Indeed, there are multiple, complex factors that impact the response to antifungal therapy, rendering it challenging to correlate in vitro susceptibility and in vivo outcomes. Such factors include the net state of immunosuppression, the pharmacokinetic and pharmacodynamics parameters affecting drug bioavailability and tissue penetration, stage of fungal disease (localized versus disseminated), and extent of surgical debridement [167]. Despite these limitations, antifungal susceptibility of *Fusarium* spp. in immunocompromised patients may still be helpful in the management of individual patients. Knowing that the MIC of a particular antifungal agent against a known hyaline mold is off-scale resistant (e.g., >16 µg/mL) likely predicts that the organism will not be effectively treated, and that an alternative agent is warranted. It is important to note that, in addition to antifungal therapy, patient outcome hinges upon the type of immunosuppression, infection site(s), extent of disease, surgical interventions, and reversal of immune impairments [168].

### 6.3. Pathogen-Directed Antifungal Therapy

#### 6.3.1. *Fusarium* Species

The in vitro MIC for all antifungal agents against Fusaria tends to be high. Amphotericin B and second-generation triazoles (voriconazole and posaconazole) are the most active agents in vitro. Isavuconazole may have more limited in vitro activity, as evidenced by a recent study of 75 clinical *Fusarium* isolates in which the MIC_50_ of isavuconazole was >16 µg/mL [169]. However, there is considerable variability in the in vitro susceptibility patterns amongst different *Fusarium* spp. and within a given species complex [170]. Fusaria are intrinsically resistant to echinocandins. As noted above, interpretive breakpoints are not defined given a lack of data correlating the in vitro susceptibility to clinical outcome; however, epidemiological cut-off values have been established [171].

For the primary therapy of invasive fusariosis, voriconazole or lipid formulation amphotericin B are recommended in the international guidelines of the European Confederation of Medical Mycology in cooperation with the International Society for Human and Animal Mycology and the American Society for Microbiology [164]. Although the absence of randomized trials in fusariosis and other hyaline mold diseases limits the strength of the treatment recommendations, the combination of in vitro, in vivo, retrospective observational, and non-randomized prospective studies provide some rational basis for guidance in the management of these uncommon mycoses. Table 2 offers a suggested approach to antifungal therapy for *Fusarium* spp. and other hyaline molds discussed in this manuscript.

Given the high mortality associated with IF and varying antifungal susceptibility by species, it is reasonable to initiate combination therapy with both agents while susceptibility data are pending. The initial report of deoxycholate amphotericin B treatment of fusariosis in patients with hematological malignancies established a clinical role for polyenes in this disease [172]. A later study of voriconazole as salvage therapy demonstrated its activity in the treatment of patients with fusariosis and other less common invasive fungal diseases [173]. At least two studies have subsequently shown improved survival in recent years with the use of either or both liposomal amphotericin B and voriconazole compared to the use of deoxycholate amphotericin B prior to the year 2000 [174,175]. Clinical data to support the use of posaconazole or isavuconazole are limited, although both have been used with success despite their higher MICs [176,177].

Reversal of immunosuppression is critical to outcome, particularly in neutropenic patients, regardless of specific antifungal regimens [48,178]. Indeed, a recent multicenter study of 88 patients with IF found that neutropenia and receipt of corticosteroids were associated with higher six-week mortality whereas mortality was similar among patients who received voriconazole, liposomal amphotericin B, or both combined [165].

#### 6.3.2. *Scedosporium* Species

Compared to *Fusarium* spp., the in vitro susceptibilities of voriconazole and amphotericin B against *Scedosporium* spp. are more predictable. Voriconazole is most active against *Scedosporium* spp., followed by posaconazole and the echinocandins [179]. High MIC values were observed for amphotericin B, isavuconazole, and fluconazole. For first-line treatment of scedosporiosis, voriconazole is recommended. This recommendation is supported by clinical studies in which immunocompromised patients with invasive scedosporiosis receiving voriconazole treatment had significantly greater survival than those receiving any formulation of amphotericin B [61,180]. Posaconazole or the combination of voriconazole and an echinocandin may be considered for salvage therapy [164]. *S. apiospermum* has intrinsic resistance to amphotericin B; therefore, the use of this agent is discouraged unless no other antifungal therapy is available [164].

#### 6.3.3. *Lomentospora prolificans*

*L. prolificans* appears to have intrinsic resistance to all approved antifungal agents, and as such, invasive disease due to this pathogen portends one of the poorest survival rates amongst the hyaline molds. Indeed, in one study, six-week mortality was 100% (11/11) among immunocompromised children and 74% (29/39) among immunocompromised adults with lomentosporiosis [61,62]. The optimal antifungal therapy is uncertain; however, recent international guidelines recommend the combination of voriconazole and terbinafine plus or minus other antifungal agents based on the in vitro data demonstrating synergy and clinical studies [164]. Supporting this recommendation for the combination of voriconazole and terbinafine are individual case reports with favorable outcome in voriconazole-resistant isolates and in vitro combination studies demonstrating synergistic interaction between voriconazole and terbinafine [181,182]. In the largest case series to date including 41 patients with invasive *L. prolificans* infection from the FungiScope registry, 28-day survival was significantly higher in those treated with voriconazole and terbinafine combination (63%) versus those receiving monotherapy (29%) [183].

Miltefosine is an alkylphosphocholine drug with in vitro activity against filamentous fungi including *L. prolificans* [184]. In combination with aggressive surgical debridement, miltefosine plus voriconazole and terbinafine have been successfully used in two patients with *L. prolificans* bone and joint infection [185,186]. More data are needed to determine if miltefosine is an effective treatment for lomentosporiosis, either as a monotherapy or in combination with other antifungal agents.

#### 6.3.4. *Scopulariopsis* Species

The optimal antifungal therapy for *Scopulariopsis* species has not yet been established as in vitro susceptibility data demonstrate markedly elevated MICs to all approved antifungal agents, and clinical studies comparing antifungal treatment regimens are lacking due to the rarity of this pathogen [10,187,188]. In vitro synergy is observed with the combinations of posaconazole and terbinafine, posaconazole and caspofungin, and amphotericin B and caspofungin; however, there are insufficient correlative animal and human data [189]. According to international guidelines, the following agents may be considered for treatment of *Scopulariopsis* infection: lipid formulations of amphotericin B, voriconazole, posaconazole, echinocandins, and terbinafine [164]. Antifungal susceptibility testing is recommended to guide the treatment decisions. However, in clinical practice, a successful outcome is rarely achieved with medical therapy alone. Rather, surgical debridement, when feasible, and the reversal of immunosuppression are critical.

#### 6.3.5. *Trichoderma* Species

*Trichoderma* species demonstrate the lowest MICs to voriconazole and echinocandins, and higher MICs to amphotericin B and posaconazole [13]. Prior to FDA approval of voriconazole, mortality approached 100% in immunocompromised patients with disseminated disease [12]. More recent data from the FungiScope registry demonstrates an overall mortality of 46% (7/15) and 28% (2/7) in patients treated with voriconazole and caspofungin, respectively, with higher rates observed in transplant recipients [11]. There are no established guidelines for the management of *Trichoderma* infection; using existing preclinical and clinical data, voriconazole alone or combined with an echinocandin is reasonable. Removal of infected peritoneal dialysis and central venous catheters and surgical debridement when feasible and applicable are important components of management.

#### 6.3.6. *Acremonium* Species

The in vitro MICs against *Acremonium* spp. for the currently approved antifungal agents are universally high with the exception of terbinafine [15,81]. Perez-Cantero and colleagues recently reviewed the literature and summarized 309 human infections due to *Acremonium*/*Sarocladium*, of which approximately one-half were invasive and one-third manifested as fungemia [14]. Of the 85 patients treated with antifungal therapy, amphotericin B was administered most often (49%), followed by voriconazole (15%) and itraconazole (13%). Due to the diverse clinical presentations and host immune status, a specific antifungal regimen could not be correlated with outcome. The 2014 European guidelines recommend voriconazole, amphotericin B, and posaconazole as potential treatment options in patients with invasive disease due to *Acremonium* species [190].

#### 6.3.7. *Paecilomyces variotii*

*Paecilomyces variotii* is resistant in vitro to voriconazole and isavuconazole, while remaining susceptible to amphotericin B, echinocandins, posaconazole, and itraconazole [88,191,192,193]. Case reports of breakthrough *P. variotii* disease in patients receiving voriconazole treatment or prophylaxis support the limited role for this agent [107]. Of note, among the 52 cases of *P. variotii* disease, there was no difference in mortality in those treated with amphotericin B versus triazoles [88]. Based upon the available in vitro and clinical data, guidelines recommend the lipid formulation of amphotericin B as first-line monotherapy and posaconazole or itraconazole for salvage treatment [164].

#### 6.3.8. *Purpureocillium lilacinum*

Whereas *P. variotii* is susceptible to amphotericin B, *P. lilacinum* is resistant. *P. lilacinum* is generally susceptible to the triazoles with the lowest observed MICs for voriconazole and posaconazole. Activity of the echinocandins is variable [89,193]. Clinical studies also suggest good outcomes when voriconazole is used for invasive *P. lilacinum* disease, and one study demonstrated improved survival in patients treated with voriconazole rather than amphotericin B [89,194]. An important component of therapy includes the removal of infected catheters or other foreign bodies when feasible [195,196]. For localized cutaneous disease, surgical debridement of infected tissue can be curative and obviate the need for adjunctive antifungal therapy [108,197]. 

#### 6.3.9. *Rasamsonia argillacea* Species Complex

Members of the *Rasamsonia argillacea* species complex are intrinsically resistant to voriconazole and isavuconazole and have high MICs of posaconazole and itraconazole. MICs/minimum effective concentrations (MEC) are low for echinocandins and variable for amphotericin B [91,198,199]. Given these susceptibility patterns and the clinical reports of breakthrough infection in CGD patients on voriconazole prophylaxis, it is recommended to avoid triazoles when treating patients with disease due to the *Rasamsonia argillacea* species complex [164,200]. Rather, primary therapy with echinocandin monotherapy or combined with lipid formulation amphotericin B or with posaconazole may be considered [164].

#### 6.3.10. *Arthrographis kalrae*

The triazoles and terbinafine have favorable in vitro activity against *A. kalrae* whereas echinocandin MICs are high and amphotericin B MICs are variable [17]. Due to a paucity of laboratory animal data and clinical experience, the optimal treatment is uncertain. However, successful treatment with itraconazole, sometimes in conjunction with surgical resection, is described in several case reports.

#### 6.3.11. *Penicillium* Species

*Penicillium* spp. are susceptible in vitro to voriconazole, posaconazole, itraconazole, amphotericin B, and caspofungin [201]. Due to its rarity as a cause of invasive fungal disease, there are limited data to inform antifungal management. Amphotericin B and its lipid formulations have been used with successful outcomes in case reports [16,202,203].

### 6.4. Role of Surgical Management

In the setting of non-pulmonary, localized infection, surgical debridement and/or drainage, when feasible, is an important component of the management of hyalohyphomycoses, particularly for pathogens demonstrating a high level of antifungal resistance such as *L. prolificans* and *Scopulariopsis* species. Surgical resection of a focal lung lesion may also be appropriate in those failing medical management or to reduce the burden of disease in patients with ongoing immunosuppression. The extent of surgical resection in craniofacial structures such as paranasal sinuses may be more conservative if coupled with optimal antifungal therapy and the augmentation of the innate host response. Resection or debridement may also be warranted for the management of osteoarticular diseases caused by the hyaline molds, as exemplified by musculoskeletal infections caused by *Scedosporium* spp. and *Lomentospora prolificans* [204]. Disseminated disease is best managed with antifungal therapy and restoration of the innate host immunity. As noted above, the removal of infected catheters and prosthetic devices is also associated with improved outcomes.

### 6.5. Strategies to Augment Host Immune Responses

Implementation of immunomodulatory treatments for invasive sino-pulmonary fungal diseases requires an understanding of the host immune responses for specific fungal pathogens, however, the host defenses against the non-*Aspergillus* hyaline molds are not well understood. However, as previously noted, some parallels may be drawn to *Aspergillus* species. For example, given the critical role of neutrophils in the pathogenesis of hyaline mold infections, granulocyte colony stimulating factor (G-CSF) and granulocyte-macrophage colony stimulatory factor (GM-CSF) may be administered to neutropenic patients who are expected to recover bone marrow function [205]. A recent study of 15 pediatric patients with hematological malignancies and refractory invasive fungal diseases as well as a literature review of 50 patients demonstrated a favorable therapeutic response rate to the administration of GM-CSF when added to antifungal therapy [206]. These results are further substantiated by prior preclinical studies that demonstrate that, in addition to recovery from neutropenia, GM-CSF augments functional oxidative and non-oxidative activities against *Aspergillus* and non-*Aspergillus* hyaline molds as well as establishing a protective effect on respiratory epithelium [44,207,208,209].

Similarly, granulocyte transfusions from dexamethasone- and G-CSF stimulated donors may have a role in the treatment of refractory invasive mold diseases in selected patients with anticipated neutrophil recovery [210,211,212]. The adoptive transfer of pathogen-specific T cells directed against *Aspergillus* spp. have also been explored for the prevention and management of invasive aspergillosis (IA); further study is needed to understand its role in both IA and non-*Aspergillus* mold diseases [213]. Interferon-γ is used for the prevention of invasive aspergillosis in patients with CGD and has been proposed as adjunctive therapy to restore immune function, as observed in a small case series of patients with IA [205,214,215]. Finally, the reversal of iatrogenic immunosuppression (e.g., reducing or discontinuing corticosteroids) is important to optimize outcomes in patients with invasive mold diseases.

Patients who develop invasive pulmonary hyaline mold infections during an initial cycle of chemotherapy-induced neutropenia are at risk of the progression or relapse of infection during subsequent cycles. For example, a patient with acute leukemia who suffers disseminated fusariosis during induction is at high risk for relapsed or progressive disseminated fusariosis during consolidation and delayed intensification [216]. In patients with hyaline mold infections in this patient population, we extended the period of recovery from neutropenia before the next cycle of chemotherapy in order to allow for neutrophil-mediated damage against hyphae. In vitro studies have demonstrated that amphotericin B formulations enhance the antifungal activity of neutrophils and monocytes against *Fusarium solani* [217]. Similarly, antifungal triazoles and polymorphonuclear leukocytes synergize to cause increased hyphal damage to *Scedosporium prolificans* and *Scedosporium apiospermum* [218].

A particularly novel and emerging area of research is the role of cancer immunotherapies as adjunctive therapy for invasive fungal diseases. Specifically, evidence that fungi induce the activation of checkpoint pathways has led to preclinical data demonstrating that the blockade of the programmed cell death protein 1 (PD-1) and cytotoxic T-lymphocyte antigen 4 pathways augments antifungal immunity and improves outcomes [219]. Thus far, clinical data are limited; a recent case report describes the long-term remission of fusariosis in a patient with acute myelogenous leukemia following the introduction of nivolimab (PD-1 inhibitor) to standard antifungal therapy [220]. 

### 6.6. Novel Investigational Therapies

Given the high levels of antifungal resistance amongst many of the hyaline molds, there is an urgent need for novel therapeutics for hyalohyphomycoses. Two investigational antifungal agents with novel mechanisms of action are currently in clinical trials: fosmanogepix (APX001; Pfizer, Inc., New York, NY, USA) and olorofim (F901318; F2G limited, Manchester, UK). 

Fosmanogepix is a first-in-class antifungal drug that inhibits Gwt1, an enzyme in the glycosylphosphatidylinositol biosynthesis pathway, compromising cell wall synthesis and leading to restricted fungal growth. This agent demonstrates potent activity against *Fusarium* spp., *Scedosporium* spp., *Lomentospora prolificans*, *Paecilomyces* spp., *P. lilacinum,* and the *Rasamsonia argillacea* species complex in vitro and/or in animal models, in addition to *Aspergillus* spp. and *Candida* species [221,222,223,224,225]. A phase 2 clinical trial of fosmanogepix for the treatment of *Aspergillus* spp. and rare molds (AEGIS) has been completed, but the results are not yet available (NCT04240886). 

Olorofim, a member of the novel antifungal class, orotomides, selectively inhibits the pyrimidine biosynthesis enzyme dihydroorotate dehydrogenase. Olorofim is unique in that it lacks activity against yeasts, but has potent in vitro activity against *Scedosporium* spp., *Lomentospora prolificans,* and the *Rasamsonia argillacea* species complex as well as *Aspergillus* spp. [226,227]. Activity against *Fusarium* spp. appears to be species specific, with higher MICs observed for the *F. solani* species complex and low MICs for the *F. oxysporum* species complex [228]. A phase 2 clinical trial for the treatment of invasive fungal infections due to resistant fungi is ongoing (FORMULA-OLS; NCT03583164). Of note, to date, there are no data on the activity of fosmanogepix and olorofim in patients with refractory neutropenia, an important area for future study [229].

## 7. Conclusions

As described herein, the non-*Aspergillus* hyaline molds are opportunistic pulmonary pathogens that primarily affect severely immunocompromised patients, but may also cause disease in immunocompetent hosts following near-drowning, trauma, and iatrogenic exposures. While certain pathogens are particularly associated with specific hosts (e.g., *Fusarium* spp. in neutropenic patients), the overlapping clinical signs/symptoms of the hyalohyphomycoses clarifies the need for microbiologic diagnosis via culture or molecular-based methods to inform management and prognosis. In the absence of large clinical trials, in vitro antifungal susceptibility testing is recommended to guide treatment decisions, and both antifungal therapy and augmentation or the restoration of innate host immune responses are critical to successful outcomes. To this end, novel antifungal agents as well as an improved understanding of the pathogenesis of the hyaline molds in relation to host immune impairment are needed to optimize the outcomes for these uncommon, yet formidable respiratory fungal pathogens.

## Figures and Tables

**Table 1:** Differential diagnosis of non-*Aspergillus* hyaline mold pathogens according to host factors and clinical manifestations.

	Neutropenia and Hematologic Malignancy	SOT and Post-Engraftment HCT Recipients	Chronic Granulomatous Disease	AIDS ^1^	Cystic Fibrosis	Immunocompetent		
						Near Drowning	Burns/ Wounds	PD-Associated Peritonitis
Relative frequency of non-*Aspergillus* hyaline molds (in rank order)	1. *Fusarium* spp. 2. *Scedosporium* spp. 3. *Lomentospora prolificans* 4. *Scopulariopsis* spp. 5. *Trichoderma* spp.	1. *Scedosporium* spp. 2. *Lomentospora prolificans* 3. *Fusarium* spp. 4. *Purpureocillium lilacinum* 5. *Paecilomyces variotii*	1. *Rasamsonia argillacea* species complex 2. *Scedosporium* spp. 3. *Lomentospora prolificans* 4. *Paecilomyces variotii*	Rare: *Scedosporium apiospermum* *Fusarium* spp. *Arthrographis kalrae* *Purpureocillium lilacinum*	1. *Scedosporium apiospermum* 2. *Lomentospora prolificans* Rare: *Rasamsonia argillacea* species complex *Arthrographis kalrae*	*Scedosporium* spp.	1. *Fusarium* spp. 2. *Scedosporium* spp. 3. *Lomentospora prolificans* Rare: *Paecilomyces variotii* *Purpureocillium lilacinum* *Scopulariopsis* spp.	1. *Purpureocillium lilacinum* 2. *Paecilomyces variotii* 3. *Trichoderma* spp.
Common clinical presentations								
Isolated sino-pulmonary disease	*Fusarium* spp. *Scedosporium* spp. *Scopulariopsis* spp.	*Fusarium* spp. *Scedosporium* spp. *Scopulariopsis* spp.	*Rasamsonia argillacea* species complex *Scedosporium* spp.	Rare	*Scedosporium apiospermum* *Lomentospora prolificans*	*Scedosporium* spp.	Rare	Rare
Fever and disseminated skin lesions	*Fusarium* spp. Rare: *Scedosporium* spp. *Acremonium* spp.	*Fusarium* spp. Rare: *Scedosporium* spp. *Acremonium* spp.	Rare	Rare	Rare	Rare	Rare	Rare
Fungemia	*Fusarium* spp. *Scedosporium* spp. *Acremonium* spp. *Lomentospora prolificans* *Purpureocillium lilacinum*	*Fusarium* spp. *Scedosporium* spp. *Acremonium* spp. *Lomentospora prolificans* *Purpureocillium lilacinum*	Rare	Rare	Rare	Rare	Rare	Rare
Central nervous system disease	*Fusarium* spp. *Scedosporium* spp. *Lomentospora prolificans*	*Fusarium* spp. *Scedosporium* spp. *Lomentospora prolificans*	Rare	Rare	Rare	*Scedosporium* spp. Rare: *Lomentospora prolificans*	Rare	Rare
Locally invasive cutaneous soft tissue infection	*Fusarium* spp. *Scedosporium* spp. *Purpureocillium lilacinum*	*Fusarium* spp. *Scedosporium* spp. *Purpureocillium lilacinum*	*Scedosporium* spp.	Rare	Rare	*Scedosporium* spp.	*Fusarium* spp. *Scedosporium* spp. *Lomentospora prolificans*	Rare

SOT: solid organ transplant, HCT: hematopoietic cell transplant, AIDS: acquired immunodeficiency syndrome. ^1^ Descriptions of non-*Aspergillus* hyaline mold infections in persons with AIDS are limited to single case reports.

**Table 2:** Suggested antifungal therapy for disease due to non-*Aspergillus* hyaline molds ^1^.

Pathogen	First-Line Antifungal Therapy	Second-Line Antifungal Therapy ^2^	Comments
*Fusarium* species	Voriconazole or LAMB	Posaconazole	Consider voriconazole plus LAMB while antifungal susceptibilities are pending Intrinsically resistant to echinocandins
*Scedosporium* species	Voriconazole	Posaconazole or voriconazole plus echinocandin	*S. apiospermum* species complex is intrinsically resistant to amphotericin B
*Lomentospora prolificans*	Voriconazole plus terbinafine	Voriconazole plus echinocandin	Resistant to all currently approved antifungal agents Strongly consider use of an investigational agent
*Scopulariopsis* species	Optimal antifungal therapy not established Combination therapy with two drugs based on susceptibility testing is recommended		Resistant to all currently approved antifungal agents In vitro synergy observed for the following drugs: posaconazole and terbinafine, posaconazole and caspofungin, and amphotericin B, and caspofungin Strongly consider the use of an investigational agent
*Trichoderma* species	Voriconazole, or voriconazole plus an echinocandin		
*Acremonium* species	Optimal therapy not established Consider voriconazole, posaconazole, or LAMB		High in vitro MICs for currently approved antifungal agents except terbinafine
*Paecilomyces variotii*	LAMB	Posaconazole or itraconazole	Resistant to voriconazole and isavuconazole
*Purpureocillium lilacinum*	Voriconazole	Posaconazole	Resistant to amphotericin B
*Rasamsonia argillacea* species complex	Echinocandin monotherapy or combined with LAMB or with posaconazole		Intrinsically resistant to voriconazole and isavuconazole
*Arthrographis kalrae*	Optimal antifungal therapy not established Initial therapy with a triazole (voriconazole, posaconazole, or itraconazole) is recommended pending susceptibility testing		
*Penicillium* species	LAMB		

LAMB: lipid formulation amphotericin B, MIC: minimum inhibitory concentration. ^1^ In addition to antifungal therapy, surgical debridement, removal of infected catheters and prosthetic devices, and reversal of immunosuppression should be considered in all patients with invasive fungal disease due to hyaline molds. ^2^ Second-line antifungal therapy should be considered for patients with refractory disease or intolerance to first-line antifungal agents.

## Data Availability

Not applicable.
